# Supplementary material for: Analysis of the Potential Range of Mountain Pine-Broadleaf Ecotone Forests and Its Changes under Moderate and Strong Climate Change in the 21st Century
Source: Plants (Basel). 2023 Oct 26;12(21):3698. doi: 10.3390/plants12213698 (PMC10648261; doi:10.3390/plants12213698)
Supplement: Supplementary file 1 [file plants-12-03698-s001.zip › plants-2613237-SI.pdf]

Table S1. The estimates of relative contributions of the environmental variables to the MaxEnt model.

Code/Unit	Variable	Percent contribution	Permutation importance
**bio10_14**	**Precipitation of Driest Month**	**31,5**	**40,4**
**bio10_4**	**Temperature Seasonality**	**28,7**	**41,6**
**srtmMaxminussrtmMin**	**Difference between maximum and mini-mum elevation**	**22,8**	**1,2**
bio10_3	Isothermality	10,3	0
**soil**	**Soil stoniness (Volumetric fraction of coarse fragments)**	**2**	**2,6**
**bio10_8**	**Mean Temperature of Wettest Quarter**	**0,8**	**0**
bio10_1	Annual Mean Temperature	0,7	2,2
**nitrogen0-5sm_res**	**Total nitrogen in the upper 0–5 cm soil layer**	**0,5**	**0,3**
**soc 0-5 wgs res max**	**Soil organic carbon content in the fine earth fraction in the upper 0–5 cm soil layer**	**0,4**	**0,3**
bio10_7	Temperature Annual Range	0,3	0,4
nitrogen15-30sm_res	Total nitrogen in the upper 15–30 cm soil layer	0,2	0,1
**bio10_6**	**Min Temperature of Coldest Month**	**0,2**	**0,1**
bio10_9	Mean Temperature of Driest Quarter	0,2	0,1
bio10_19	Precipitation of Coldest Quarter	0,2	2,9
bio10_2	Mean Diurnal Range	0,2	0,1
ocs 0-30 wgs res max	Organic carbon stocks in the upper 0–5 cm soil layer	0,2	0
nitrogen5-15sm_res	Total nitrogen in the 5–15 cm soil layer	0,1	0,1
**bio10_15**	**Precipitation Seasonality**	**0,1**	**7,4**
bio10_16	Precipitation of Wettest Quarter	0,1	0
soc5-15 wgs res max	Soil organic carbon content in the fine earth fraction in the 5–15 cm soil layer	0,1	0
cec_0-5cm_res	Cation exchange capacity in the upper 0–5 cm soil layer	0,1	0
bio10_12	Annual Precipitation	0,1	0
nitrogen30-60sm_res	Total nitrogen in the 30–60 cm soil layer	0	0,2
bio10_11	Mean Temperature of Coldest Quarter	0	0,1
strm_max	AltitudeMax	0	0
bio10_5	Max Temperature of Warmest Month	0	0,1
strm_min	AltitudeMin	0	0
bio10_13	Precipitation of Wettest Month	0	0
strm mean	AltitudeMean	0	0
cec_5-15cm_res	Cation exchange capacity in the 5–15 cm soil layer	0	0
bio10_18	Precipitation of Warmest Quarter	0	0
bio10_17	Precipitation of Driest Quarter	0	0
cec_30-60cm_res	Cation exchange capacity in the 30–60 cm soil layer	0	0
bio10_10	Mean Temperature of Warmest Quarter	0	0
cec_15-30cm_res	Cation exchange capacity in the 15–30 cm soil layer	0	0

Note: Ecological predictors left after removal of predictors with high pair correlation are marked in bold.

Table S2. Correlation analysis of the environmental variables.

	bio01	bio02	bio03	bio04	bio05	bio06	bio07	bio08	bio09	bio10	bio11	bio12	bio13	bio14	bio15	bio16	bio17	bio18	bio19	Srtm min	Srtm mean	Srtm min-max	Srtm max	soil	nitro-gen 0-5	soc 0-5
o02	-0,88																									
bio03	-0,94	0,97																								
bio04	-0,70	0,91	0,86																							
bio05	1,00	-0,86	-0,92	-0,66																						
bio06	0,99	-0,93	-0,97	-0,79	0,98																					
bio07	-0,67	0,90	0,84	0,99	-0,63	-0,76																				
bio08	-0,06	0,03	0,00	0,13	-0,06	-0,09	0,10																			
bio09	0,99	-0,93	-0,97	-0,80	0,98	1,00	-0,76	-0,07																		
bio10	1,00	-0,87	-0,93	-0,68	1,00	0,98	-0,64	-0,06	0,98																	
bio11	0,99	-0,93	-0,97	-0,79	0,98	1,00	-0,76	-0,09	1,00	0,99																
bio12	-0,48	0,25	0,33	0,05	-0,50	-0,41	0,00	-0,07	-0,44	-0,50	-0,43															
bio13	-0,75	0,56	0,63	0,30	-0,77	-0,70	0,26	0,03	-0,70	-0,77	-0,71	0,82														
bio14	-0,33	0,12	0,19	0,02	-0,33	-0,27	-0,04	-0,13	-0,31	-0,34	-0,29	0,91	0,56													
bio15	-0,59	0,59	0,61	0,42	-0,61	-0,61	0,43	0,15	-0,57	-0,60	-0,59	0,04	0,57	-0,33												
bio16	-0,78	0,58	0,66	0,33	-0,79	-0,73	0,29	0,01	-0,74	-0,79	-0,74	0,86	0,99	0,63	0,51											
bio17	-0,21	-0,01	0,06	-0,11	-0,21	-0,14	-0,16	-0,16	-0,18	-0,22	-0,16	0,88	0,48	0,99	-0,42	0,55										
bio18	-0,78	0,59	0,66	0,34	-0,80	-0,73	0,30	0,03	-0,74	-0,80	-0,74	0,86	0,99	0,62	0,52	1,00	0,54									
bio19	0,19	-0,40	-0,34	-0,47	0,17	0,27	-0,51	-0,21	0,23	0,17	0,24	0,68	0,19	0,84	-0,64	0,24	0,91	0,24								
srtmmin	-0,97	0,92	0,96	0,80	-0,96	-0,99	0,77	0,08	-0,99	-0,97	-0,99	0,44	0,70	0,31	0,55	0,73	0,18	0,73	-0,23							
srtmmean	-0,99	0,92	0,96	0,79	-0,98	-1,00	0,75	0,07	-1,00	-0,98	-1,00	0,46	0,71	0,32	0,56	0,75	0,19	0,75	-0,21	0,99						
srtmminmax	0,41	-0,44	-0,44	-0,42	0,40	0,43	-0,41	0,07	0,44	0,41	0,43	-0,10	-0,22	-0,05	-0,19	-0,24	0,00	-0,24	0,18	-0,53	-0,45					
srtmmax	-0,98	0,92	0,95	0,79	-0,97	-0,99	0,75	0,10	-0,99	-0,98	-0,99	0,46	0,72	0,33	0,57	0,75	0,20	0,76	-0,21	0,98	0,99	-0,35				
soil	0,30	-0,29	-0,26	-0,29	0,29	0,32	-0,28	-0,14	0,31	0,30	0,31	0,09	-0,06	0,15	-0,22	-0,07	0,19	-0,07	0,29	-0,34	-0,31	0,48	-0,26			
nitrogen0-5	0,52	-0,63	-0,60	-0,66	0,51	0,58	-0,65	-0,09	0,57	0,51	0,57	-0,06	-0,26	-0,01	-0,34	-0,28	0,08	-0,28	0,32	-0,59	-0,57	0,42	-0,56	0,20		
soc0-5	0,44	-0,62	-0,57	-0,66	0,42	0,51	-0,67	-0,07	0,51	0,42	0,50	-0,04	-0,22	0,02	-0,32	-0,24	0,10	-0,24	0,32	-0,52	-0,51	0,39	-0,49	0,13	0,87	
ocs_0-30	0,75	-0,76	-0,77	-0,77	0,74	0,79	-0,72	-0,25	0,79	0,75	0,79	-0,23	-0,47	-0,14	-0,47	-0,49	-0,02	-0,50	0,33	-0,80	-0,79	0,41	-0,79	0,36	0,68	0,56
